# A perspective for sequencing familial hypercholesterolaemia in African Americans

**DOI:** 10.1038/npjgenmed.2016.12

**Published:** 2016-05-11

**Authors:** Michelle L Wright, David Housman, Jacquelyn Y Taylor

**Affiliations:** 1 Department of Primary Care, School of Nursing, Yale University, Orange, CT, USA; 2 Biology Department, Massachusetts Institute of Technology, Cambridge, MA, USA

## Abstract

African Americans suffer disproportionately from poor cardiovascular health outcomes despite similar proportions of African Americans and Americans of European ancestry experiencing elevated cholesterol levels. Some of the variation in cardiovascular outcomes is due to confounding effects of other risk factors, such as hypertension and genetic influence. However, genetic variants found to contribute to variation in serum cholesterol levels in populations of European ancestry are less likely to replicate in populations of African ancestry. To date, there has been limited follow-up on variant discrepancies or on identifying variants that exist in populations of African ancestry. African and African-American populations have the highest levels of genetic heterogeneity, which is a factor that must be considered when evaluating genetic variants in the burgeoning era of personalised medicine. Many of the large published studies identifying genetic variants associated with disease risk have evaluated populations of mostly European ancestry and estimated risk in other populations based on these findings. The purpose of this paper is to provide a perspective, using familial hypercholesterolaemia as an exemplar, that studies evaluating genetic variation focused *within* minority populations are necessary to identify factors that contribute to disparities in health outcomes and realise the full utility of personalised medicine.

## Introduction

A recent article postulates that race should be eliminated from genetic research in favour of focusing on ancestry.^1^ We agree that race, is a primarily social construct that if used for genetic research has the potential to perpetuate ineffective treatment in the clinical setting, and that using ancestry informative makers for genetic analysis is required for robust analysis that replicates between studies. However, there has been a general lack of inclusion of ethnic minority populations in studies evaluating the genetic contribution to complex disease aetiology^2^ despite known significant genetic variation by ancestry.^3,4^ Furthermore, African Americans suffer disproportionately from a variety of poor health outcomes, from preterm birth^5^ to cardiovascular disease,^6^ and neither social nor genetic variation independently explain these disparities.^7–11^ The crux of the issue we wish to elucidate is that the genetic variation specifically within African-American populations that contribute to disparities in health outcomes, like cardiovascular disease, is largely unrecognised and understudied. Here we will describe a gap in knowledge regarding genetic markers related to blood lipid levels and familial hypercholesterolaemia (FH) as an exemplar of limited understanding of the genetic variation that may contributes to health disparities within the African-American population.

## Familial hypercholesterolaemia

FH is an autosomal dominant disorder that is characterised by extremely high levels of low-density lipoprotein cholesterol resulting in cholesterol deposits in tendons and premature cardiovascular disease. The frequency of FH is now believed to be ~1 in 200 in most populations.^12^ More than 90% of mutations that cause FH are loss of function mutations in the low-density lipoprotein receptor (*LDLR*) gene (OMIM # 143890); and most of the remaining cases have mutations in apolipoprotein B (*APOB*, OMIM # 107730) or proprotein convertase subtilisin-like kexin type 9 (*PCSK9*, OMIM # 603776). However, the number of individuals identified with FH in most populations, including the United States, is only ~1% of affected individuals. This is a particularly serious lapse in screening since ~2% of sudden myocardial infarction deaths in males under 50 and females under 60 are the consequence of heterozygous FH and are preventable with statin therapy.^12^

A working group of cardiologists recently made a consensus recommendation emphasising the urgent worldwide need for diagnostic screening for FH.^12^ Populations in Norway and the Netherlands are screened, diagnosed and treated at a rate between 43–71%, while screening in other nations generally remains below 1% and it is unclear how may individuals of African ancestry are screened.^12^ These alarmingly low numbers of individuals screened for FH, particularly those not of European ancestry, places many at risk from early morbidity and mortality unnecessarily. Furthermore, it should be noted that most countries do not collect information on the prevalence of FH and estimates for various populations, not including those of African ancestry, were made based on European populations for the recommendations developed for FH screening.

As described by Yudell *et al.*,^1^ using race as a proxy in genetic studies and to evaluate disease risk may lead to the incorrect assumption that certain populations are unaffected or do not carry certain genetic risk variants. FH is a tangible example of this particular issue. Indeed African Americans suffer from FH, although African Americans that present with FH are less likely to have a known FH pathogenic variants associated with their condition identified when they have diagnostic sequencing.^13,14^ In fact, 77% of African Americans that presented with autosomal dominant hypercholesterolaemia did not carry any of the known risk alleles in the three genes commonly associated with FH.^13^ This suggests that genetic risk variants for FH in populations of African ancestry are different than those of European ancestry and have not yet been identified. It is unclear whether studies evaluating FH in populations of African ancestry have not been completed or if they were excluded for other reasons.

## Genetic contribution of variation in serum lipid levels

Nearly one third of adults in the United States have elevated cholesterol levels and almost half of the people affected have total cholesterol levels above 240 mg/dl.^15^ Less than half of affected individuals are receiving treatment; and of those who are, only one third have their condition under control.^16^ African-American populations consistently have lower total cholesterol and triglyceride levels and higher high-density lipoprotein levels (‘good’ cholesterol) relative to other ethnicities in the United States, but approximate those of European ancestry.^16,17^ Worldwide, African populations have the lowest burden of hypercholesterolaemia of all global regions and African Americans may share some variants that have a protective role.^18,19^ However, the higher high-density lipoprotein levels observed in African Americans does not confer the same protective effect related to heart disease as seen in other populations because African Americans continue to experience earlier onset of cardiovascular-related disease and suffer worse outcomes.^7^ These outcomes could be related to the higher prevalence of other cardiovascular risk factors in African Americans, such as hypertension and obesity, although ancestry is a potential confounder.

African and African-American populations have the highest levels of genetic heterogeneity,^20^ which is a factor that must be considered in the burgeoning era of personalised medicine. For example, in previous genome-wide association studies (GWAS) that evaluated the association between serum cholesterol and triglycerides levels with single-nucleotide polymorphisms (SNPs), SNPs that were significant in a population of European ancestry were less likely to replicate in those of African ancestry compared with Asian and East Asian populations.^21^ Some of this discrepancy may be due to variation in allele frequencies between populations of European and African ancestry. Of the 163 SNPs that were found to be significantly associated with altered serum cholesterol and triglycerides in two GWAS studies,^21,22^ 30 of the SNPs minor allele frequencies are reversed (i.e., the minor in European populations is the major allele in African populations) and 15 SNPs have minor allele frequency of <5% in African-American populations (i.e., they are less prevalent in African than European ancestry populations). Previous studies estimate that ancestry informative genetic markers in African Americans more closely approximate populations from West Africa (71%) than Europe (13%), although the percentage of African ancestry is highly variable within the African-American population.^20^ This variability in percentage of African and European ancestry in African Americans likely contribute to poor identification/replication of genetic markers associated with disease risk, because many of the large genomic studies have been completed in cohorts representing mostly European ancestry populations. Conversely, genetic variation associated with altered serum cholesterol levels have also been associated with improved immunity in African populations.^19^ This is a prime example why variation within minority populations should be studied further, because some genetic variants associated with evolutionary advantage in one geographic location may not be as advantageous as in another.

Variation in individual serum cholesterol levels are also known to be polygenic.^21,23^ Differences in combinations of contributing alleles could also contribute to the lower levels of replication in genetic studies between populations not of European or mixed ancestry. For example, significant SNPs from GWAS studies^21,22^ located within genes associated with FH have similar allele frequencies in African Americans (ASW) and European Americans (CEU) populations (rs6511720 (LDLR ASW=0.12, CEU=0.1), rs2413395 (APOB ASW=0.17 CEU=0.33), and rs2479409 (PCSK9 ASW=0.32 CEU=0.34); www.1000genomes.org). These particular SNPs are not listed as pathogenic variants for FH, but contribute to variation in serum cholesterol levels. Therefore, genomic variation for FH among African Americans may be different than that seen among European Americans, thereby necessitating further examination of FH among this understudied population. If scientists do not further explore the variation of FH among African-American populations, many people with this genetic predisposition may be missed and suffer grave clinical consequences unnecessarily.

## Future directions

The value of genetic screening of adults for pathological genetic variants, such as FH that cause serious pathological conditions, has been demonstrated in principle, for a significant number of conditions.^24^ A barrier to the implementation of genetic screening programmes is the cost/benefit ratio for each individual programme. Due to this, cascade screening has been successfully implemented in European countries to successfully identify a number of individuals with FH.^12,25^ This approach to identifying affected individuals occurs once an individual is diagnosed with FH, who becomes the proband, and subsequent family members are screened based on relationship and medical history. However, cascade testing is less effective when few probands for a condition are known, such is the case for FH in the United States.^26^ In addition, cascade screening results in identification of FH status at a much later age, and treatment should begin as early as childhood for homozyogotes and heterozygotes with aggressive FH presentation.^25,26^ Subsequently, the marked reduction in cost of whole-genome sequencing (WGS) now makes it possible to reduce the cost of sequence based screening for pathological variants causing many different conditions by amortising the cost of each condition screened as a fraction of the total sequence cost. It is anticipated that WGS cost will drop further over the next decade making the economics of WGS even more attractive for this use compared with individual sequence panels for a subset of genes relevant to a particular condition. WGS could be particularly useful in identifying FH variants that are specific for populations of African ancestry that have not yet been identified.^13^

Most genetic studies to date have viewed population structure as a problem to be avoided in WGS, but with improved analysis methods, we are interested in conducting case–control studies within different ethnic groups as a way to increase power and discover variants that influence phenotypes. Power to detect a genetic variant that influences disease depends crucially on the allele frequency of the variant, and different populations have different allele frequencies due to demographic forces (e.g., due to either genetic drift or natural selection). Others have developed variations of ‘trans-ethnic’ meta-analysis approaches to better identify associations of causal variants with phenotypic differences by capitalising on variations in linkage disequilibrium by ancestry.^27–31^ Wang *et al*.^30^ found trans-ethnic approaches that utilise a Bayesian framework^27^ or more relaxed assumptions in a random-effect model^31^ identify potential causal variants better that traditional GWAS meta-analysis approaches in populations not of European ancestry. However, Asimit *et al*.^29^ argue that including more participants of African ancestry in the initial study design out performs statistical meta-analysis approaches in fine mapping of genetic associations in populations not of European ancestry.

In analysis of rare variant effects on disease, such as FH, it is essential to consider that these variants are likely to be population specific and/or geographically restricted.^32–38^ For example, a common variant that influences diabetes risk in Greenland increases disease risk 10-fold, but is essentially absent in the rest of the world.^39^ This variant could be identified with a smaller sample size than usually used in genetic studies because of its large effect. Furthermore, utilising this approach will enable us to also identify variants that may have protective health effects in populations of African ancestry, as we have found with other cardiovascular conditions.^40^ We suggest using African ancestry cohorts and comparing results with diverse cohorts to search for African ancestry population-specific strong effects that influence health outcomes. Given that inclusion of more participants with African ancestry improves fine-mapping mapping of causal variants,^29^ this may be the best approach to identify variants specific to populations of African ancestry that are unknown.

## Conclusion

Both environmental and genetic factors contribute to disparities in health outcomes, although limited studies strive to identify genetic variants within the African-American community that contribute to poor outcomes. The genetic heterogeneity within the population results in some overlap with multiple populations. However, there may be variants unique to this population that have not been identified or polygenic variants that are unique to African-American populations due to mixed ancestry. Personalised medicine aims to improved health outcomes and in order to accomplish this goal, more studies evaluating genomic variants related to disease—both known and unknown—are direly necessary. We recommend that trans-disciplinary scientists teams (nurses, physicians, genetic counsellors and biostatisticians) mine GWAS and genome sequencing studies with African-American participants to further explore differences within African Americans for FH in order to truly understand the genomic underpinnings of disease process in order to devise targeted individualised therapeutics that will be beneficial in reducing health disparities and eliminating disease.^23^ Further genomic studies, particularly genome sequencing based, that target larger numbers of African Americans are warranted in order to advance understanding of FH disease process for improved treatment and health outcomes.
